# Association between exogenous testosterone use and new diagnosis of hidradenitis suppurativa: A case series

**DOI:** 10.1016/j.jdcr.2023.11.012

**Published:** 2023-11-25

**Authors:** Kanika Kamal, Najiba Afzal, Amina Ziad, Alexandra Charrow

**Affiliations:** a Department of Dermatology, Brigham and Women’s Hospital, Boston, Massachusetts; b Harvard Medical School, Boston, Massachusetts; c School of Medicine, University of California, Davis, Sacramento, California

**Keywords:** androgen replacement therapy, exogenous testosterone, hidradenitis suppurativa, hypogonadism

## Introduction

Hidradenitis suppurativa (HS) is a chronic, inflammatory skin condition presenting with painful nodules, abscesses, and sinus tracts, typically distributed near apocrine sweat glands and initially presenting in the peripubertal period.^1^ Although the condition has a female predominance, HS research to date suggests that androgens may drive the disease for some, as selected patients with HS exhibit higher concentrations of total testosterone than nonaffected controls.^2^ Additionally, medications that lower testosterone either directly or indirectly (oral contraceptives, spironolactone, and finasteride) are well established treatments for milder HS.^3^

Exogenous testosterone prescriptions for gender affirmation,^4^ hypogonadism, or low libido,^5^ are increasingly commonplace. Yet, little is known about how exogenous testosterone, including various routes of administration and dosing, may affect the risk of developing HS, with only one case reported in the literature to date.^6^ Here, we highlight and discuss all cases of new HS diagnoses which developed in a single major academic center over a 6 year period following initiation of exogenous testosterone for hypogonadism or decreased sexual desire.

## Methods

We queried the Mass General Brigham Research Patient Data Registry from November 2016 to November 2022 and identified all patients with a dermatologist-verified diagnosis of HS and a prescription of exogenous testosterone indicated only for decreased sexual desire or hypogonadism and compared against all patients who did not have a dermatologist-verified diagnosis of HS but did receive a prescription of exogenous testosterone indicated only for decreased sexual desire or hypogonadism in the same time frame. The exogenous testosterone routes of administration included in this study are testosterone propionate gel (AndroGel), which is typically applied to the upper portion of the arms and shoulders, transdermal patches (Androderm), powder, subcutaneous pellet implants (Testopel), and testosterone propionate or cypionate intramuscular injections (Depo-Testosterone). Two raters (K.K. and N.A.) reviewed each patient chart and recorded type of exogenous testosterone, dose, and frequency, as well as date of first mention and severity of physician-diagnosed HS. Only patients given a diagnosis of HS within 12 months of starting exogenous testosterone were included in this case series. We excluded all patients with a diagnosis or mention of HS, abscesses, nodules, sinus tracts, or boils in their chart before starting exogenous testosterone.

## Results

A total of 27 patients met the inclusion criteria for this study. Out of 27 patients, 3 patients were identified who developed new HS within 12 months of starting exogenous testosterone (Table I) for the indications of interest. The remaining 24 patients had developed HS prior to initiation of exogenous testosterone. Two (66.7%) patients identified as White and 1 (33.3%) as Asian. Mean age (SD) age was 53 (10.5) years. Two (66.7%) identified as cisgender men and 1 (33.3%) as a cisgender woman. Intramuscular Depo-Testosterone was initiated in 1 (33.3%) patient, whereas the other 2 (66.7%) were prescribe topical exogenous testosterone (AndroGel and Micronized Powder) (Table II).

**Table I:** Patients prescribed exogenous testosterone with and without diagnosis of HS

Total patients who were prescribed exogenous testosterone for hypogonadism/decreased sexual desire WITH a diagnosis of HS	3
Total patients who were prescribed exogenous testosterone for hypogonadism/decreased sexual desire WITHOUT diagnosis of HS	27

*HS*, Hidradenitis suppurativa.

**Table II:** Demographic and clinical characteristics of 6 patients with new dermatologist-verified diagnosis of hidradenitis suppurativa after starting exogenous testosterone

Pt	Age (decade)	Gender identity	Race/ethnicity	ET indication	Initial ET formulation	ET dose/frequency	Time to HS diagnosis	Hurley stage at diagnosis	Change to ET post-HS diagnosis?	Biologic management for HS?	Disease progression, ED visits, or hospitalizations?
1	40s	Cisgender man	Asian	Hypogonadism/low libido	Topical (AndroGel)	20.25 mg/1.25 g (1.62%) twice a day	12 mo	Stage I	Y, started 200 mg intramuscular ET biweekly 15 mo after HS diagnosis	N	N
2	60s	Cisgender man	White	Hypogonadism/low libido	Intramuscular (Depo-Testosterone)	200 mg biweekly	12 mo	Stage I	Y, reduced to 100 mg intramuscular biweekly 15 mo after HS diagnosis	N	N
3	50s	Cisgenderwoman	White	Hypogonadism/low libido	Topical compounded	1 mL/100 mg, application 1 inch daily	5 mo	Stage I	Y, began implant (Testopel) 75 mg daily release 10 mo after HS diagnosis	N	N

*ED*, Emergency department; *ET*, exogenous testosterone; *HS*, hidradenitis suppurativa; *Pt*, patient.

All 3 patients (100%) were diagnosed with Hurley stage I HS. The average time to diagnosis of HS after starting exogenous testosterone was approximately 10 months (SD 4.0). Location of HS involvement was unrelated to site of administration of exogenous testosterone.

After new diagnosis of HS, 2 patients (33.3%) changed the formulation of their exogenous testosterone from topical (AndroGel and Micronized Powder) to intramuscular and implantable Testopel, respectively. One patient (patient 3) reduced the dose of his intramuscular exogenous testosterone from 200 mg to 100 mg biweekly. None of the patients stopped exogenous testosterone therapy at any point after HS diagnosis.

Regarding HS treatment, all 3 patients (*n* = 3, 100%) started first-line agents for mild HS, including oral antibiotics and topical washes. From the time to diagnosis to present, no patients had evidence of disease progression, emergency room visits, or hospitalizations for HS.

## Discussion

In this case series, we present 3 patients in a 6-year period who developed new HS within 12 months of starting topical or intramuscular exogenous testosterone therapy. Although the role of exogenous testosterone in causing HS is not known in these cases, HS is thought to begin with occlusion of the follicular pilosebaceous unit.^1^ Excess androgens, which are known to regulate hair growth and sebum production in this unit,^7^ may be mediating worsening of the disease through excess sebum production or increased occlusion of the follicular unit. Androgen effects on HS may also be mediated through its role in worsening insulin resistance or by changing gut and skin microbiome over time.^8^ Finally, androgens have known effects on fat and muscle distribution which may in turn worsen HS for some patients as well.^8^

There are limited cases of new HS in patients using exogenous testosterone for any indication and exogenous testosterone is an increasingly common prescription.^9^ Critical to determining the role of androgen in HS is better understanding those whose disease is sensitive to it and why. Larger cohort studies are needed to better elucidate the factors that may make some individuals more susceptible to androgen mediated HS initiation and exacerbation.

This study has limitations. First, we only included patients who presented with HS within 12 months of starting exogenous testosterone therapy. Given that the typical time from presentation of lesions to formal diagnosis of HS is 10 years, this 12-month period may capture not the time to symptoms, but rather the time to availability of dermatology appointments or the time to access health care.^10^ Alternatively, it may be possible that some patients had HS-like lesions before starting exogenous testosterone, but experienced an exacerbation after initiating exogenous testosterone that led them to present to dermatology. Other limitations include a small sample size and representation from a single institution.

In conclusion, the findings of this case series highlight the complex interplay between hormonal factors and HS pathogenesis. Future observational and case-control research studies are needed to explore the relationship between exogenous androgens and HS, elucidate underlying mechanisms, and guide treatment strategies for affected patients.

## Conflicts of interest

Dr Charrow serves on the Advisory Board for Novartis and UCB and has advised for Q32. Authors Kamal, Afzal, and Ziad have no conflicts of interest to declare.
